# Assessment of SARS-CoV-2 Infection among Healthcare Workers of a German COVID-19 Treatment Center

**DOI:** 10.3390/ijerph18137057

**Published:** 2021-07-01

**Authors:** Lionel Larribère, Jelizaveta Gordejeva, Lisa Kuhnhenn, Maximilian Kurscheidt, Monika Pobiruchin, Dilyana Vladimirova, Maria Martin, Markus Roser, Wendelin Schramm, Uwe M. Martens, Tatjana Eigenbrod

**Affiliations:** 1 Cancer Center Heilbronn-Franken, SLK Clinics Heilbronn GmbH, 74078 Heilbronn, Germany; Dilyana.Vladimirova@slk-kliniken.de (D.V.); Uwe.Martens@slk-kliniken.de (U.M.M.); 2 GECKO Institute for Medicine, Informatics and Economics, Hochschule Heilbronn, 74081 Heilbronn, Germany; jgordeje@stud.hs-heilbronn.de (J.G.); maximilian.kurscheidt@hs-heilbronn.de (M.K.); monika.pobiruchin@hs-heilbronn.de (M.P.); wendelin.schramm@hs-heilbronn.de (W.S.); 3 Institute of Laboratory Medicine, SLK Clinics Heilbronn GmbH, 74078 Heilbronn, Germany; lisa.kuhnhenn@slk-kliniken.de (L.K.); markus.roser@slk-kliniken.de (M.R.); tatjana.eigenbrod@slk-kliniken.de (T.E.); 4 Institute for Infection Prevention and Clinical Hygiene, SLK Clinics Heilbronn GmbH, 74078 Heilbronn, Germany; Maria.Martin@slk-kliniken.de

**Keywords:** COVID-19, SARS-CoV-2, epidemiology, infectious disease, health care workers, transmission, infection risk

## Abstract

To date, more than 160 million people have been infected with COVID-19 worldwide. In the present study, we investigated the history of SARS-CoV-2 infection among 3067 healthcare workers (HCW) in a German COVID-19 treatment center during the early phase of the pandemic (July 2020) based on the seroprevalence of SARS-CoV-2 antibodies and self-reported previous PCR results. The results demonstrate a low prevalence of SARS-CoV-2 infection (*n* = 107 [3.5%]) with no increased risk for employees with a high level of patient exposure in general or working in COVID-19-confined areas in particular. This suggests that the local hygiene standards implemented in our hospital during the first wave of COVID-19 pandemic were effective in preventing patient-to-HCW transmission. No evidence for highly mobile staff serving as a vector for SARS-CoV-2 transmission could be found. In addition, impairment of smell and/or taste was strongly associated with SARS-CoV-2 history.

## 1. Introduction

As of mid-May 2021, the World Health Organization (WHO) has confirmed a total of more than 160 million cases of coronavirus disease 2019 (COVID-19) and more than 3 million related deaths around the world [1]. In Germany, the pandemic has affected more than 3.5 million people by now and has led to at least 86,000 deaths [2]. Apart from causing considerable acute disease burden and mortality, the COVID-19 pandemic has also significant psychological consequences, causing anxiety, depression, sleeping disorders or physical symptoms including headaches, temporomandibular disorders or back pain [3,4,5].

In this context, healthcare systems and especially public and private hospitals play a particular role: healthcare workers (HCW) are at the frontline, in daily contact with COVID-19 patients and are therefore potentially at a higher risk than the global population [6,7,8]. New hygiene regulations and standards based on the WHO guidelines were adopted by the German federal government and local authorities to prevent patient-to-HCW transmission. These standards were implemented in our institution for staff working with COVID-19 patients and included the usage of FFP2 masks, isolation gowns, gloves and face shield or goggles. Due to a shortage of personal protective equipment (PPE) during the early phase of the pandemic, the usage of PPE in our hospital was initially restricted to HCW in direct contact with COVID-19 patients, either in the emergency room (ER) or on COVID-19 wards. FFP2 masks sometimes had to be re-used during several shifts and protective gowns had to be worn for more than one patient. A shortage of PPE especially in the early phase of the pandemic, resulting in unprotected contacts of HCWs with COVID-19 patients, has been reported worldwide [4,9,10]. Only in autumn 2020 did FFP2 masks become gradually available in our hospital for all employees. However, data on whether employees with a high level of exposure to COVID-19 patients might be at a greater risk of infection despite the usage of PPE are controversial [8,11,12,13]. Moreover, the role of housekeeping and transport staff with high mobility within the hospital as a potential vector of SARS-CoV-2 transmission remains poorly investigated.

The SLK Clinics are a large healthcare provider with approximately 5000 employees; it became a COVID-19 treatment center in southwest Germany. More than 800 patients tested positive for SARS-CoV-2 in our hospital between 1 March and 30 June 2020. The surveillance data provided by the Robert Koch Institute (German government agency responsible for identification, surveillance and prevention of diseases) demonstrate that the highest incidence of SARS-CoV-2 infections in Germany occurred in March and April 2020 and eased by May/June, corresponding to the first wave of COVID-19 [14]. In late summer, the incidence of SARS-CoV-2 rose again, culminating in a steep increase in October 2020 and delineating the beginning of the second wave.

In July 2020, when the first COVID-19 wave had eased and automated serological immunoassays became available, we investigated the cumulative incidence of SARS-CoV-2 infection among employees according to their different functions and departments based on the seroprevalence of SARS-CoV-2 antibodies and self-reported previous PCR results. The goal of this study was to determine a potential correlation between working conditions in a German COVID-19 treatment center and evidence of SARS-CoV-2 history in order to evaluate risk factors for COVID-19 infection. Our data show that the overall prevalence of SARS-CoV-2 history in our hospital was low (3.5%) and did not depend on the work area (COVID-19-confined versus regular units). Employees with high in-house mobility did not serve as a potential disease vector.

## 2. Materials and Methods

### 2.1. Study Design and Setting

All employees aged ≥ 14 years were invited to participate in this study on a voluntary basis. The trial has been registered at the German Clinical Trial Register (Deutsches Register Klinischer Studien, identification number DRKS00022226T). All participants provided a written informed consent. In case of adolescents aged ≤ 18 years (mostly nursing students and trainees), informed consent was provided by the legal guardian. Blood samples were obtained between 1 July and 21 July 2020. No specific inclusion or exclusion criteria were applied. Back in summer 2020 no participants were enrolled in a COVID-19 vaccine study. Participants were asked to fill out a paper-based standardized questionnaire including questions on age, gender, work area, contact to (COVID-19) patients, in-house mobility, previous SARS-CoV-2 PCR testing and symptoms of illness compatible with SARS-CoV-2 infection between January 2020 and June 2020. Questionnaires were transcribed into an electronic form stored in a separate section of the hospital’s information system. Questionnaires of participants with a positive PCR or serology result were independently double checked by a second reviewer to ensure high data quality. Serum samples from 3088 employees and 3067 matched questionnaires were obtained. CSV-based exports of the laboratory results and electronic questionnaires were imported into a study database (PostgreSQL 13.1 [15]) to enable further data analyses.

### 2.2. Antibody Testing

Serum samples were analyzed for the presence of SARS-CoV-2 antibodies as part of a complementary study comparing the performance of five different commercially available immunoassays. Therefore, serum samples were tested in up to five different assays detecting either SARS-CoV-2 anti-S or anti-N antibodies. Specifically, all 3067 serum samples were analyzed using the SARS-CoV-2 Total assay detecting S1 IgG/IgM on the automated ADVIA Centaur immunoassay system (Siemens; Munich, Germany). All samples tested positive on Centaur as well as a random selection of samples tested negative on Centaur were further analyzed with four additional automated immunoassays: Architect SARS-CoV-2 IgG (N-antigen) (Abbott; Chicago, IL, USA), Liaison SARS-CoV-2 S1/S2 IgG (Diasorin; Saluggia, Italy), Anti-SARS-CoV-2 S1 IgG (Euroimmun; Lübeck, Germany) and Anti-SARS-CoV-2-NCP IgG (Euroimmun; Lübeck, Germany).

### 2.3. Definition of SARS-CoV-2 History

**Figure 1:**
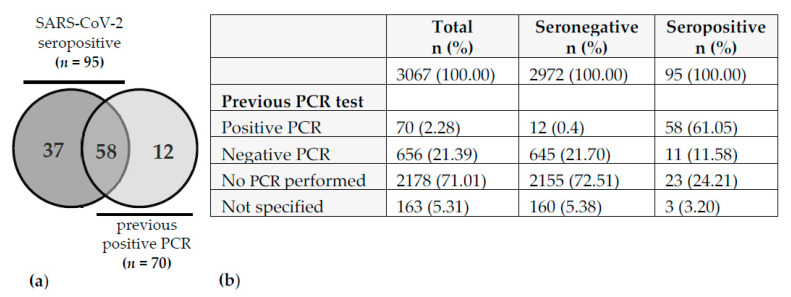
Distribution of previous SARS-CoV-2 PCR tests and serostatus. (**a**) Venn diagram represents 107 cases with SARS-CoV-2 history. (**b**) Table depicts the distribution of PCR tests in the whole cohort (*n* = 3067).

Positive history of SARS-CoV-2 was defined by the presence of specific antibodies in the serum and/or by a self-reported previous positive PCR test. Seropositivity was defined as follows: ≥ 2 tests positive (*n* = 92) or 1 test positive plus ≥ 1 test borderline (*n* = 3, all three employees self-reported a previous positive PCR from naso-/oropharyngeal swab). Therefore, the total number of seropositive samples was 95. Since 12 participants reported a positive PCR test without having SARS-CoV-2 specific antibodies in the serum, the total number of participants with a positive SARS-CoV-2 history was 107 (Figure 1).

### 2.4. Statistical Analysis

Data were analyzed with the statistics software R version 3.6.3 [16]. Logistic regression analysis was performed to determine whether age, gender, in-house mobility, working from home, contact to (COVID-19) patients, and type of jobs are influencing factors for a positive SARS-CoV-2 status. Because of the binary outcome of the model, McKelvey & Zavoina Pseudo R^2^ (M & Z Pseudo R^2^) [17] was calculated to assess the goodness of fit. Implementation of M & Z Pseudo R^2^ was provided by the R package DescTools version 0.99.41 [18]. One participant was excluded from the logistic regression analysis due to an implausible answer given in the questionnaire (thus the total number of analyzed samples was *n* = 3066). A second logistic regression model was calculated to determine whether certain clinical symptoms are associated with a positive SARS-CoV-2 status (*n* = 3067).

## 3. Results

The serum samples with matched study questionnaires from 3066 employees obtained between 1 July and 21 July 2020 were included in this analysis. The characteristics of the study cohort are summarized in Table 1. The gender distribution was 80.3% females and 19% males (0.7% of the participants did not state their gender). Overall, 107 (3.5%) employees with a history of SARS-CoV-2 were identified (Figure 1). As mentioned above, SARS-CoV-2 history was defined by seropositivity and/or by a self-reported previous positive PCR. No correlation with age could be found. However, male employees had a slightly higher risk of SARS-CoV-2 infection (Table 1).

Out of the 107 participants with SARS-CoV-2 history, 95 were seropositive, of which 58 (61%) also stated a previous positive PCR. Interestingly, 12 out of 70 (17%) employees with a reported positive PCR result in the past did not show detectable anti-SARS-CoV-2 antibodies at the time of the study (maximum 5 months period between positive PCR test and serological analysis) (Figure 1).

In the next step, potential occupational risk factors for COVID-19 infection were evaluated (Table 1).

Although performed on a voluntary basis, this study included approximately 60% of all employees and covered all work areas. The goodness of fit measures for the logistic regression model on occupational risk factors yielded an M & Z Pseudo R^2^ of 0.85. However, we could not find significant factors. This discrepancy will be addressed in the discussion section. Neither working from home compared to on-site work nor different levels of in-house mobility had a significant impact on the risk of infection. Moreover, no significant differences in the history of SARS-CoV-2 infection were observed between physicians, nursing staff/medical assistance/non-medical therapists and administrative staff. Employees working in the laboratory, in pathology or pharmacy as well as housekeeping and transport staff showed a tendency towards a lower infection risk, yet data were not significant due to the small number of cases. Thus, we do not have evidence that employees with high mobility within the hospital served as a vector of SARS-CoV-2 transmission. Of note, employees working in IT and technical services tended to have a higher risk of COVID-19 infection (3.10 [0.98; 9.68], *p* = 0.05). In this particular case, we identified an infection cluster in the IT department, in which the index person caused at least three consequent cases. Moreover, at least two employees in this cluster had a confirmed infection of private origin. 2040 out of 3066 participants (66.5%) reported a high level of direct patient contact (>50% of their activity). Moreover, 1135 of all participants (37%) reported specifically working with COVID-19 patients either on a COVID-19-related ward or in the ER. Nevertheless, we could not find a higher prevalence of COVID-19 history for employees who had a general contact with patients or for employees working on COVID-confined units and/or in the ER. Therefore, different levels of exposure to patients in general or to COVID-19 patients specifically did not affect the COVID-19 infection risk.

Finally, we assessed the correlation of reported COVID-19 compatible symptoms with history of SARS-CoV-2 (Table 2). According to odds ratio (OR) ranking, the strongest associated symptom was the impairment of smell and/or taste. This symptom had a positive predictive value (PPV) of 35.9% for COVID-19 infection. Other positively correlated symptoms were: apathy, weight loss, loss of appetite, abnormal sleepiness/drowsiness, fever ≥ 38 °C, shortness of breath, respiratory distress, malaise/weakness and muscle pain. The M & Z Pseudo R^2^ for the model on clinical symptoms was 0.16. Very few SARS-CoV-2 infected participants (4.7%) stated having no symptoms between January and June 2020 as compared to 24.8% of SARS-CoV-2 negative individuals.

## 4. Discussion

In this study, we assessed the history of SARS-CoV-2 infection among HCWs in a German hospital after the first wave of the COVID-19 pandemic. Our results show a cumulative incidence of 3.5% for SARS-CoV-2 infections. This number is rather low although a little higher than in the general population of our region, Baden-Württemberg, where a seroprevalence of 1.8% in adults was reported during a similar time frame [19]. Our results are in line with other studies conducted in German hospitals, indicating a low prevalence of SARS-CoV-2 specific antibodies in HCWs [20,21,22]. For example, a seroprevalence of 3.5% was reported in HCWs with direct patient contact in a medical center in Southern Germany in April 2020 [23]. Similarly, the study of Korth et al. conducted in HCWs at a German University Hospital reported a seroprevalence of 2.2% in March–May, which increased to 4.0% in June–July and to 5.1% in October–December 2020 [24,25].

Interestingly, we observed a lack of detectable antibody levels in 17% of participants with a self-reported PCR-based diagnosis of COVID-19. This is in line with other studies in which approximately 10–22% of PCR-confirmed COVID-19 cases were negative in serology more than 14 days after diagnosis [26,27,28,29]. As the sensitivity of the Siemens Centaur SARS-CoV-2 total antibody test has been reported as 98.1–100% (information provided by the manufacturer and [30]), it seems unlikely that all of these cases were missed due to an insufficient sensitivity of the assay. It is rather conceivable that these participants either did not seroconvert at all or developed only low antibody titers, which had already disappeared at the time of our study. Thus, our findings support the accumulating evidence that SARS-CoV-2 antibodies rapidly decrease after infection, especially in patients with mild COVID-19 symptoms [31,32,33,34,35]. It is therefore probable that the cumulative incidence in our study, as well as in other studies, is slightly underestimated.

Our data do not support an increased risk for SARS-CoV-2 infection in HCWs with high levels of patient exposure in general or to COVID-19 patients in particular. Moreover, we did not find evidence that certain job categories were more affected than others or that highly mobile staff served as a vector for SARS-CoV-2 transmission. The lack of statistically significant risk factors for infection may partially be explained by the small sample sizes in certain categories. However, we assume that this does not greatly affect the main conclusion of our study. Indeed, when pooling all groups with contact to COVID-19 patients, the prevalence of SARS-CoV-2 history was 3.52% compared to 3.55% of employees with no contact to COVID-19 patients. These findings are in line with other publications from Germany and other European countries. For example, a longitudinal study performed in more than 1000 employees in a hospital in northern Germany detected SARS-CoV-2-specific antibodies in 4.3% of participants but did not identify patient care as risk factor for seropositivity [20]. Similarly, a study conducted in a tertiary care center in Belgium did not find an association between serostatus and involvement in clinical care in general or specifically in care for COVID-19 patients in more than 3000 participants with an overall seroprevalence of 6.4% [36]. In a large hospital in Spain, cumulative prevalence of SARS-CoV-2 infection was reported with 11.2%, yet neither the professional category nor daily patient contact or working in COVID-19 units was identified as a risk factor for infection [37]. However, some hospitals indeed reported a higher SARS-CoV-2 prevalence for example in housekeeping staff or employees with high exposure to (COVID-19) patients [8,11,38,39]. For example, a study conducted in more than 3000 employees of a large health care provider in Sweden and Denmark identified the number of patient contacts during a workday as the most prominent predictor for seropositivity (2.8% in Denmark and 8.3% in Sweden). In this study, ambulance staff had the highest risk of seropositivity, which might be explained by either a high level of interaction with patients or by the fact that they cannot reject patients with possible symptoms of COVID-19 [7]. In the same line, general practitioners were the category of physicians with the highest number of deaths in Italy, presumably due to the high number of patient contacts in conjunction with an insufficient access to PPE especially at the beginning of the pandemic [10]. In addition, Weinberger et al. reported that HCW on both COVID-19 and regular non-COVID-19 wards had a greater rate of seroconversion than non-frontline personnel. Moreover, both the intensity and number of risk contacts were associated with seropositivity in their study [8].

Yet, as already indicated, data on this topic remain controversial. Since the onset of the COVID-19 pandemic, a large number of studies on SARS-CoV-2 seroprevalence among HCWs has been published in Europe and worldwide. In a recent systematic review, Vaselli et al. comprehensively summarize the results of 53 studies investigating the seroprevalence among HCWs in 13 European Countries between February 2020 and August 2020. These data demonstrate a strong heterogeneity with seroprevalence rates ranging from 0.7% to 45.3% in HCWs across Europe [40]. While the majority of studies reported a seroprevalence of <10% during the indicated time period, only a few studies (mostly conducted in the UK) described seropositivity rates between 20–45%. However, due to the pronounced heterogeneity of the data, Vaselli et al. did not find significant differences in the seroprevalence amongst HCWs when stratified by country. Likewise, the risk of exposure to COVID-19 patients did not have a statistically significant impact on the seroprevalence, although all subgroups with a reported seroprevalence of >30% had been categorized as high or medium risk exposure [40]. It is likely that the different numbers obtained in various studies are highly dependent on the region and the overall SARS-CoV-2 prevalence but also on differences in public health strategies. Moreover, infection clusters originating from undiagnosed infected patients or staff within the hospital as well as failure to adhere to infection control measures can significantly impact the seroprevalence in HCWs in different hospitals.

In this context, an important question is the origin of infection among the employees in our hospital. A retrospective retrieval of these data in our study cohort was not possible as this information was not part of the questionnaire. In addition, *n* = 37 out of 107 (34.6%) participants with COVID-19 history did not report a previous positive PCR (Figure 1) and were therefore not aware of their infection, thus making it impossible to retrospectively assess the origin of infection. Nevertheless, the hospital’s Department of Infection Prevention and Control routinely contacts employees with a positive PCR and investigates the most likely source of infection. The first case of a positive SARS-CoV-2 PCR detected in an employee of the SLK Clinics was registered at the end of February 2020 after a vacation in Italy. By the end of June 2020, the Department of Infection Prevention and Control had registered 70 PCR-positive employees which matches the number of participants with a self-reported positive PCR in our study. They were either identified in the in-house testing offered to all employees or tested elsewhere and informed the hospital of their positive test result. All positive employees were interviewed to investigate the most plausible origin of infection. In only 9 out of 70 cases (12.9%), infections were attributed to patient-to-HCW transmission. Five of these employees were involved in an outbreak at the very beginning of the pandemic in March 2020. The low rate of patient-to-HCW transmission is in line with a recent, sequencing-based investigation demonstrating that only 4.2% of COVID-19 infections in HCWs in a US academic medical institution could be traced to a patient contact [41]. The small proportion of infections that were attributed to patient-to-HCW transmission in our study suggests that the hygiene measures applied at SLK Clinics during the first wave of the COVID-19 pandemic were effective. Yet, the rather low transmission rates in our study also go along with the only moderate secondary infection rate of household members of a COVID-19 index person that has been reported with approximately 16% [42,43].

The vast majority of SARS-CoV-2 infections in our HCWs during the first wave of COVID-19 most likely originated from private contacts outside the hospital (*n* = 34/70; 48.6%) or from contacts with positive colleagues (*n* = 24/70; 34.3%). In three cases, the origin of infection remained unclear as there was no determinable contact to a COVID-positive person. The high proportion of HCW-to-HCW transmission might be explained by the fact that the obligation to wear surgical masks for employees to reduce the risk of transmission was implemented for patient-related areas only at the end of March 2020 and for all staff only in early summer 2020 when surgical masks became available in sufficient quantities. Our findings are also supported by data from Celebi et al. who identified that staying in the same break room with another HCW without wearing a medical mask for more than 15 min as well as failure to keep a safe social distance from colleagues were risk factors for COVID-19 infection [44]. Although physical distancing is an important component in limiting the spread of COVID-19, Keller et al. identified restricted space in break rooms or work areas, the need to communicate confidential patient information or the wish to maintain relationships at work as barriers to adhering to these rules [45]. That HCW-to-HCW transmission or especially contacts outside the hospital might be a more relevant source of infection in HCWs has also been appreciated by others [36,41,46,47].

In our study, very few participants (4.7%) with history of COVID-19 reported having had no symptoms between January and June 2020, as compared to 24.8% of SARS-CoV-2 negative individuals. The number of asymptomatic individuals was thus much lower than in other studies reporting more than 20% of asymptomatic infections [26,29,42]. The reason for this discrepancy is unclear but might partially be explained by the fact that our study evaluated symptoms within a relatively large time frame. Thus, the reported symptoms may have not occurred at the same time as the COVID-19 infection.

Finally, we found a strong association between the impairment of smell and/or taste with SARS-CoV-2 history. Indeed, a strong association of that symptom with COVID-19 has already been described in other studies [4,48,49,50,51]. Nevertheless, it is known that the symptom of taste and smell impairment is not pathognomonic of COVID-19 and has also been reported for influenza and common cold [52,53]. This could explain the fact that we observed a high OR for this symptom but a PPV of only 35.9%. In addition, all reported COVID-19-associated symptoms so far are rather unspecific and can occur in a variety of viral infections. Moreover, COVID-19 cannot be pinpointed to a single symptom rather than a group of symptoms. This is one possible explanation for the relatively low M & Z Pseudo R^2^ for this model. Future research might include the application of clustering algorithms to determine which symptoms occur together more frequently.

Our study has several limitations: relatively few participants were identified with history of SARS-CoV-2 (*n* = 107 positives vs. *n* = 2960 negatives), causing a class imbalance of positive vs. negative sub-groups. This imbalance could produce statistical artifacts and may account for the high M & Z Pseudo R^2^ (0.85) in the model for the occupational risk factors. Indeed, the discrepancy between a high Pseudo R^2^ and not finding significant predictors is not entirely unexpected. The lack of significant occupational risk factors for infection may partly be due to the small sample sizes in certain categories. We decided not to retrospectively pool certain data to achieve higher numbers and fewer categories as this may introduce statistically significant findings but may also result in too heterogeneous groups. Nevertheless, we assume that these limitations do not greatly affect the main conclusions of our study. For instance, the fact that we did not find a statistically significant increase of risk between low (<10%) and high (>50%) levels of general patient exposure is supported by the observation that patient-to-HCW transmission seemed to play a minor role in our hospital. Another limitation is the fact that our questionnaire did not evaluate in which medical field the participants were working. An analysis whether certain medical categories might be more affected than others, as recently reported for example for acute and general internal medicine as well as for family doctors [10,39], was therefore not possible.

## 5. Conclusions

In conclusion, we report a low prevalence of SARS-CoV-2 history (3.5%) in the employees of a large German hospital with specialized COVID-19 treatment units. The risk of infection in employees did not depend on their work area (COVID-19-confined versus non-confined wards) and employees with high in-house mobility did not serve as a potential disease vector.

## Figures and Tables

**Table 1:** Association between history of SARS-CoV-2 infection and sociodemographic or working characteristics. CI: confidence interval; OR: odds ratio; Significance codes (SC): ≤0.001—***/≤0.01—**/≤0.05—*; Intercept: OR [95% CI] = 0.02 [0.00;0.12], *p*-Value = 0.00045.

	Negative n (%) ^#^	Positive n (%) ^#^	OR [95% CI]	*p*-Value	SC
**Age**					
14–19 (Ref. Group)	48 (1.6)	1 (0.9)			
20–29	515 (17.4)	19 (17.8)	1.87 [0.36;34.29]	0.55090	
30–39	660 (22.3)	27 (25.2)	2.12 [0.42;38.77]	0.47152	
40–49	627 (21.2)	21 (19.6)	1.81 [0.36;33.20]	0.56863	
50–59	830 (28.1)	32 (29.9)	2.26 [0.45;41.07]	0.43346	
≥60	279 (9.4)	7 (6.5)	1.38 [0.23;26.47]	0.76833	
**Gender**					
Female (Ref. Group)	2377 (80.3)	76 (71.0)			
Male	562 (19.0)	31 (29.0)	1.77 [1.05;2.92]	0.02735	*
Not specified	20 (0.7)	-	0.00 [-]	0.99133	
**Working from Home**					
No (Ref. Group)	2753 (93.0)	95 (88.8)			
Yes	168 (5.7)	9 (8.4)	1.67 [0.75;3.29]	0.16849	
Not specified	38 (1.3)	3 (2.8)	2.86 [0.65;8.77]	0.10124	
**In-house Mobility**					
<10% (Ref. Group)	1297 (43.8)	48 (44.9)			
10–50%	294 (9.9)	4 (3.7)	0.73 [0.44;1.20]	0.22103	
>50%	1958 (66.2)	82 (76.6)	1.00 [0.59;1.65]	0.98574	
Not specified	40 (1.4)	-	1.57 [0.24;5.69]	0.55710	
**Patient Contact**					
<10% (Ref. Group)	667 (22.5)	21 (19.6)			
10–50%	294 (9.9)	4 (3.7)	0.37 [0.10;1.11]	0.10087	
>50%	1958 (66.2)	82 (76.6)	1.08 [0.47;2.65]	0.85662	
Not specified	40 (1.4)	-	0.00 [-]	0.98755	
**Work Area**					
Physicians (Ref. Group)	445 (15.0)	17 (15.9)			
Administration, patient management, reception	356 (12.0)	10 (9.3)	0.81 [0.27;2.38]	0.69788	
Housekeeping and transport services	186 (6.3)	1 (0.9)	0.17 [0.01;0.90]	0.09369	
IT and technical services	73 (2.5)	9 (8.4)	3.10 [0.98;9.68]	0.05174	
Laboratory, pathology and pharmacy staff	111 (3.8)	-	0.00 [-]	0.98017	
Nursing staff, medical assistance and non-medical therapists	1629 (55.1)	69 (64.5)	1.35 [0.74;2.57]	0.33705	
Others/not specified	159 (5.4)	1 (0.9)	0.21 [0.01;1.16]	0.14371	
**Contact COVID-19 Patients**					
No (Ref. Group)	1818 (61.4)	67 (62.6)			
Yes, COVID-19 suspect ward	260 (8.8)	12 (11.2)	1.08 [0.54;2.00]	0.80862	
Yes, combinations of COVID-19 wards and ER	353 (11.9)	11 (10.3)	0.66 [0.32;1.26]	0.23625	
Yes, COVID-19 ICU	215 (7.3)	5 (4.7)	0.46 [0.16;1.08]	0.10652	
Yes, COVID-19 ward	102 (3.4)	2 (1.9)	0.47 [0.08;1.55]	0.29777	
Yes, ER	165 (5.6)	10 (9.3)	1.38 [0.64;2.73]	0.37505	
Not specified	46 (1.6)	-	0.00 [-]	0.98642	

^#^ Analysis was performed on a total of participants *n* = 3066 (1 exclusion due to an implausible answer given in the questionnaire).

**Table 2:** History of SARS-CoV-2 infection in participants and association with COVID-19 associated clinical symptoms. CI: confidence interval; OR: odds ratio; Significance codes (SC): ≤0.001—***/≤0.01—**/≤0.05—*; Intercept: OR [95% CI] = 0.05 [0.04;0.06], *p*-Value = 0.00000.

	Negative n (%)	Positive n (%)	OR [95% CI]	*p*-Value	SC
Headache (Ref.Group)	1298 (43.9)	65 (60.7)			
Fever ≥ 38 °C	338 (11.4)	51 (47.7)	3.01 [2.04; 4.42]	0.00000	***
Impairment of taste/smell	100 (3.4)	56 (52.3)	11.18 [7.41; 16.89]	0.00000	***
Congested/running nose	1161 (39.2)	51 (47.7)	0.88 [0.60; 1.27]	0.49358	
Cough	958 (32.4)	52 (48.6)	1.08 [0.74; 1.57]	0.67286	
Sore throat/hoarseness	1204 (40.7)	47 (43.9)	0.78 [0.53; 1.14]	0.20292	
Shortness of breath	296 (10.0)	35 (32.7)	2.36 [1.52; 3.61]	0.00009	***
Respiratory distress	88 (3.0)	11 (10.3)	2.50 [1.21; 4.72]	0.00786	**
Abnormal sleepiness/drowsiness	64 (2.2)	10 (9.3)	3.12 [1.45; 6.11]	0.00172	**
Apathy	31 (1.0)	11 (10.3)	7.09 [3.28; 14.35]	0.00000	***
Loss of appetite	130 (4.4)	27 (25.2)	4.15 [2.52; 6.66]	0.00000	***
Weight loss	53 (1.8)	13 (12.1)	4.90 [2.45; 9.20]	0.00000	***
Stomach pain	291 (9.8)	6 (5.6)	0.41 [0.16; 0.88]	0.03977	*
Diarrhea	453 (15.3)	19 (17.8)	0.84 [0.48; 1.38]	0.50587	
Nausea/vomit	230 (7.8)	11 (10.3)	0.96 [0.47; 1.76]	0.89041	
Joint pain	508 (17.2)	37 (34.6)	1.45 [0.95; 2.19]	0.07789	
Muscle pain	459 (15.5)	40 (37.4)	1.74 [1.15; 2.61]	0.00778	**
Swollen lymph nodes	135 (4.6)	6 (5.6)	0.89 [0.34; 1.93]	0.78441	
Conjunctivitis	76 (2.6)	7 (6.5)	1.84 [0.75; 3.89]	0.14194	
Skin rash	106 (3.6)	7 (6.5)	1.32 [0.54; 2.76]	0.50025	
Malaise/weakness	634 (21.4)	61 (57.0)	1.92 [1.34; 2.76]	0.00041	***
Other symptoms	51 (1.7)	9 (8.4)	3.52 [1.57; 7.15]	0.00101	**
No symptoms since 1 January 2020	734 (24.8)	5 (4.7)	0.14 [0.05; 0.31]	0.00002	***
Not specified	111 (3.8)	2 (1.9)	0.36 [0.06; 1.17]	0.15838	

## Data Availability

The raw data are available upon reasonable request from the corresponding author.
